# L-arabinose and D-xylose: sweet pentoses that may reduce postprandial glucose and insulin responses

**DOI:** 10.29219/fnr.v65.6254

**Published:** 2021-07-23

**Authors:** Korrie Pol, Monica Mars

**Affiliations:** 1 Wageningen University & Research, Division of Human Nutrition and Health, Wageningen, The Netherlands

**Keywords:** L-arabinose, D-xylose, glycaemic response, insulinaemic response, sucrase inhibition, randomized controlled trial

## Abstract

**Background:**

Diets inducing high fluctuations in plasma glucose levels are linked to type 2 diabetes. L-arabinose and D-xylose have been hypothesized to inhibit intestinal sucrase activity, delay sucrose digestion, and reduce glycaemic and insulinaemic responses. However, few human studies have assessed this using realistic foods.

**Objective:**

We investigated the effects of the addition of L-arabinose and D-xylose on glucose homeostasis using a fruit-based drink and the effect of L-arabinose using a muffin.

**Design:**

Fifteen males participated in two double-blind, randomized cross-over experiments. In experiment A, three drinks were tested: (1) L-arabinose, (2) D-xylose and (3) control drink. In experiment B, two muffins were tested: (1) L-arabinose and (2) control muffin. All products consisted of ~50 g available carbohydrates, and L-arabinose or D-xylose was added as 10% of sucrose. Pre- and post-ingestive plasma glucose and insulin levels were measured at fixed time points up to 180 min after consumption.

**Results:**

Glucose and insulin peaks were lower after the L-arabinose and D-xylose drink than the control drink (*P* < 0.01). After consumption of the muffin, glucose responses were not significantly different; however, the insulin peak and incremental area under the curve (iAUC) tended to be lower for the L-arabinose muffin.

**Conclusion:**

L-arabinose and D-xylose are functional ingredients that can potentially lower the post-ingestive glycaemic and insulinaemic responses when added to realistic foods. However, the efficacy of applying L-arabinose appears to depend on the food matrix. Addition of these compounds needs further testing in other foods and in other populations, such as pre-diabetics.

## Popular scientific summary

- Fruit-based drinks and muffins are examples of realistic food products to which functional ingredients can be added.
- This randomized controlled trial investigated the effects of L-arabinose and D-xylose on glucose homeostasis using a fruit-based drink and the effect of L-arabinose using a muffin in healthy male subjects.
- L-Arabinose and D-xylose are functional ingredients that can potentially lower the post-ingestive glycaemic and insulinaemic responses when added to realistic food products, but the efficacy depends on the food matrix.

Glycaemic control is critical in the context of metabolic disease, such as diabetes and impaired glucose tolerance. It has been shown that an increased intake of foods with a low impact on glucose responses, such as high-fibre foods, is associated with a lower prevalence of type 2 diabetes, coronary heart disease and their associated health risk markers (1–3). Moreover, there are indications that foods low in glycaemic response might affect appetite (4), although this effect depends on many factors and has yet to be proven causally (5).

Sweet tasting carbohydrates that have a low impact on the glycaemic response may be healthier alternatives to sucrose. An ingredient that is currently of interest is L-arabinose, a pentose that can be derived from plant materials by enzymatic hydrolysis, such as hemicellulose from sugar beets (6). Hemicellulose is naturally abundant in plant material, such as arabic gum and sugar beets (6). L-arabinose has a sweet taste (7) and can, therefore, be easily added to sweet-tasting foods, such as beverages and muffins, without compromising the overall taste. Another ingredient of interest is D-xylose, a sweet pentose that can be extracted from cell walls in the hemicellulose backbone of cereal grains and wood (8). L-arabinose and D-xylose share a common functionality, namely, the inhibition of sucrose hydrolysis in the brush border of the small intestine by uncompetitive inhibition of the enzyme sucrase (9–11). An advantage of ingesting L-arabinose or D-xylose together with sucrose may, therefore, be delayed digestion of sucrose, and consequently, a slower absorption of glucose. This is then followed by delayed and decreased glucose and insulin responses (9, 12). The advantage of adding L-arabinose and D-xylose to sucrose-rich products is that taste is not compromised, and foods may be similarly liked by consumers.

So far, only a handful of human intervention studies have investigated L-arabinose and glucose homeostasis (10, 13–16). Most of these studies found that the addition of L-arabinose to sucrose in water exerted a dampening effect on postprandial blood glucose and insulin responses (10, 13, 14, 16). The dosages in these studies, investigating effects in drinks and gels, ranged from 0.9 g L-arabinose per 30 g sucrose (13) to 15 g L-arabinose per 35 g sucrose (16). Similar effects were found when D-xylose was added to sucrose and water solutions (8, 12, 17). However, the effect of adding L-arabinose to realistic food products, containing realistic amounts of sucrose, has not been thoroughly investigated.

Up to now, little data are available on the effects of L-arabinose on blood glucose responses of matrix effects in real-life products. To our knowledge, only two previous studies have added L-arabinose to realistic foods: one of the studies using buns and muffins (15) and, more recently, the effect of replacing sucrose with L-arabinose in grinded cereals was investigated (16). The results of these two studies were inconsistent, and thus, additional studies using realistic food applications are warranted. In this research study, we provide knowledge on implementing addition of L-arabinose in real-life food products rich in sucrose to enhance blood glucose control, as this information is currently lacking. The main objective of the current study was to compare the glycaemic and insulinaemic responses in human subjects to L-arabinose added to realistic liquid and solid sucrose-containing food products, namely, fruit-based drinks and muffins. We hypothesized that as matrices become more complex, more L-arabinose is needed, and therefore, we choose to add 10% w/w L-arabinose, which is a higher dosage than previous studies used. Thus, in the current study, realistic food products were tested, having a realistic amount of sucrose (i.e. 50 g in drinks and 25 g in muffins) and L-arabinose (10% of sucrose), in order to mimic real-life foods that could safely contribute to lowering glycaemic responses. The secondary objective was to compare these effects with D-xylose added to the fruit-based drink. We also monitored subsequent *ad libitum* energy intake and potential effects on appetite and wellbeing. Furthermore, to explore the underlying mechanism of the biological functionality of L-arabinose and D-xylose, we investigated sucrose metabolism in more detail. For this, we used the recovery of ^13^C-labelled sucrose measured in exhaled CO_2_. This measurement is a proxy for the rate at which the glucose molecule derived from sucrose is absorbed into the bloodstream and metabolised in the liver. We hypothesized that in addition to dampening of the plasma glucose and insulin response, we may observe a slower recovery of ^13^C after ingestion of sucrose together with L-arabinose or D-xylose.

## Materials and methods

### Subjects

We recruited healthy male volunteers in April 2015 in Wageningen (The Netherlands) and from surrounding areas. For the recruitment, emails were sent to persons included in a database of volunteers who had previously expressed interest in the participation in nutrition studies. Eligibility criteria included the following: male, healthy (as judged by the subject), age 18–35 years, normal body weight (Body Mass Index (BMI) 18.5–25 kg/m^2^), stable body weight (had not reported weight loss or weight gain of >5 kg in the 2 months prior to the screening session), normal fasting glucose (fasting glucose < 6.1 mmol/L, as measured by finger prick) and normal haemoglobin concentration (fasting Hb > 8.5 mmol/L, as measured by finger prick). Exclusion criteria were as follows: inability to communicate in spoken and written Dutch, medicine use, any known diseases, allergy or intolerance to the food products used in the study, excessive alcohol consumption (≥21 glasses/week on average) and excessive physical activity.

We aimed to include a total of 18 subjects in this study. Sample size calculations showed that at least 13 subjects were needed to detect a 10% difference in the blood glucose peak between treatments. In this study, we estimated that the variation coefficient would be 15% in blood glucose peak levels in our population (homogeneous population; only male, small age and BMI range). This was based on the research that used similar techniques to compare the addition of L-arabinose and D-xylose to sugar water with a control and observed a 10% difference in the glucose peak (10, 12). For sample size calculations, we set the power at 0.80, α = 0.05, and a correlation within subjects of 0.7 (two-sided paired *t*-test). The power and sample size calculations were performed with G*Power v3.0.10 (University of Düsseldorf). To take into account dropouts and missing data, we included an additional five subjects in this study.

After recruitment, receipt of oral and written information, and signing of the consent form, 37 men completed an inclusion questionnaire. In addition, they completed the Dutch Eating Behaviour Questionnaire (DEBQ) to identify eating restraint, emotional eaters and external eaters (18). In preliminary analyses, it was found that none of these personality traits affected glucose or insulin responses; hence, they will not be further discussed. Out of these 37 subjects, 25 were found to be eligible. Subjects fasted prior to arrival to the research site for clinical measurements, including measurement of height (Seca Ltd, UK), bodyweight (Seca Ltd., UK) and a finger prick blood sample to determine Hb and fasting blood glucose concentrations. Body weight and height were measured to the nearest 0.1 kg and 0.5 cm, respectively. After the initial visit, seven subjects could not participate in this study due to planning and personal reasons (*n =* 3), too low Hb (*n =* 1), medication use (*n =* 2) or antibiotic use (*n =* 1). In total, 18 subjects were enrolled in this study. Fifteen subjects successfully completed the trial and were included in the analysis (Table 1). During the trial, three subjects dropped out after the second test day due to planning (*n =* 1), procedure (*n =* 1), and a combination of planning and procedure (*n =* 1). The subjects did not significantly differ in age and BMI from those who dropped out or were excluded. During the trial, subjects did not experience any adverse events.

**Table 1:** Subject characteristics of the 15 men that completed the study. All values are mean ± SD

Characteristics		Range
Age, years	23.4 ± 3.0	19–30
Smoking	*n =* 3	
Non-smoking	*n =* 12	
Body weight, kg	76.5 ± 7.8	65.8–96.8
Height, m	1.85 ± 0.06	1.77–1.97
BMI, kg/m^2^	22.3 ± 1.6	19.1–24.9
Restrained eating score^*^	1.76 ± 0.70	1.00–3.10
Emotional eating score^*^	1.93 ± 0.56	1.23–3.00
External eating score^*^	3.27 ± 0.53	2.40–4.30

* Measured by using the Dutch Eating Behaviour Questionnaire, range: 1 = low to 5 = high (18).

The protocol was approved by the METC-WU (ABR No.: NL51738.081.15) and was conducted according to the guidelines laid down in the 1964 Declaration of Helsinki and its later amendments. A written informed consent was obtained from all subjects. The trial was pre-registered in a public database (NL4818/NTR5319, the Netherlands Trial Register, www.trialregister.nl). Subjects received financial compensation for their participation.

### Design

The study was a double-blind randomized, cross-over trial with five treatments, including a within-block design, that is, drinks and foods. Subjects were randomized first to three drinks (experiment A) and then to two muffins (experiment B). An independent research assistant generated a list with randomized sequences by balanced block randomisation using Statistical Analyses Software (SAS) software version 9.3 (SAS Institute Inc., Cary, NC, USA). The researchers allocated the included subjects in consecutive order to the randomised sequences. The products were coded at the manufacturers’ site. Subjects and researchers were blinded within blocks.

The drinks tested in experiment A were as follows: (1) control drink, (2) D-xylose drink and (3) L-arabinose drink. The products tested in experiment B were (1) control muffin and (2) L-arabinose muffin. The drinks were studied in the first 3 weeks (experiment A) and the solids in the last 2 weeks (experiment B). In order to minimize carry-over effects, there was a washout period of at least 7 days between treatments.

### Test foods

All test foods contained ~50 g available carbohydrates (Table 2). The drinks were fruit-based drinks with a tropical fruit flavour of 500 mL, with 42 g sucrose per serving. The drinks had D-xylose or L-arabinose added as ~10 wt% of sucrose. One portion of muffins was 115 g, and contained 24 g sucrose and 2.4 g L-arabinose (~10 wt% of sucrose). All test foods were stored frozen until the afternoon before the test day, when the drinks or muffins were then taken out of the freezer and defrosted at room temperature. The drinks were stored in the fridge until the test day. All test foods were labelled with ^13^C-sucrose (see the section on ^13^C isotope recovery for more details on the labelling).

**Table 2:** Description and nutrient composition of the fruit-based drinks and muffins

	A. Fruit-based drinks (FBD)			B. Muffins	
	FBD	FBD + Ara	FBD + Xyl	Muffins	Muffins + Ara
Energy (kJ/100 g)	180	198	188	1,697	1,700
Dry matter (%)	10	10	10	77	77
Available Carbohydrates (g)	48.5	48.5	49.0	49.9	51.3
Starch (g)	-	-	-	23.3	22.9
Sucrose (g)	42.0	38.0	38.5	24.7	24.2
D-xylose (Xyl) (g)	-	-	3.5	-	-
L-arabinose (Ara) (g)	-	3.5	-		2.4
Fat (g)	-	-	-	25.5	25.0
Protein (g)	-	-	-	6.8	6.7
Fibre (g)	-	-	-	1.3	1.2
Total weight (g)	500	500	500	115	115
Drinking water (g)	-	-	-	135	135

Products were specially developed for this study by Royal Cosun. Nutrient content of the fruit-based drinks was analysed at an independent laboratory and reported here. Nutrient content of the muffins was calculated based on the recipe provided by the producer. Nutrient values were estimated by means of the Dutch Food Composition database (NEVO-online versie 2019/6.0, RIVM Rijksinstituut voor Volksgezondheid en Milieu, Bilthoven, Nederland). L-arabinose and D-xylose were assumed to be available carbohydrates and to provide 17 kJ/g.

### Experimental procedure

#### Prior to testing

In order to standardize fasting state, subjects received a frozen evening meal (fried rice or couscous) to eat the evening before the test day. They were asked to choose one type of meal after the screening, and every week they received the same meal. These meals were similar in nutrient composition and energy content (43en% carbohydrates, 22en% protein, 35en% fat and 520 kJ/100 g). Subjects were instructed to prepare their meal according to the instructions in a microwave and not to add, for example, any fats or sauces. Each pre-packed meal weighed 800 g, and subjects were instructed to eat until they were comfortably full. A diary was distributed to all subjects to fill out on the day before the test day. We asked the subjects to note down how much of the meal was leftover. Moreover, subjects filled out the time of consumption of the meal, whether they ate any product naturally rich in ^13^C (e.g. corn containing foods), if they experienced side effects or other physical complaints, if they used medication or if they had any other remarks. The day prior to testing, subjects were asked to avoid alcohol and strenuous exercise, not to eat after 20.00 h and not to drink any calorie-containing drinks after 22.00 h the evening prior to the test.

#### Test day

After a 9–12 h overnight fast, subjects arrived at the study centre between 7.30 and 8.00 h. They handed in their diary and received a new one for the coming test day. Furthermore, they filled out a well-being questionnaire and an appetite questionnaire, and breath samples were taken in duplicate. A cannula was then inserted into a forearm vein by an experienced research nurse, and a fasted venous blood sample was obtained. This sample was used as the baseline measurement.

The first three test days, subjects received the drinks and drank it with a straw. The last two test days, subjects received the muffins. Subjects were instructed to consume the products gradually over a 5-min period. After the muffins were consumed, subjects drank 135 mL of water. They remained seated as much as possible throughout the experimental session. Blood and breath samples, and appetite questionnaires were collected at baseline and at 15, 30, 45, 60, 90, 120 and 180 min after the subjects began consuming the test product. Appetite questionnaires continued to be collected at 75, 150, 240 and 270 min. After 120 min, subjects drank 135 mL of water. Four hours (240 min) after the start of consumption of the test food, an *ad libitum* lunch was served.

### Collection and handling of blood samples

Venous blood samples for glucose and insulin analyses were collected in 4 mL ethylenediaminetetraacetetic acid (EDTA) vacutainers and kept in ice water for a maximum of 15 min before being centrifuged. The tubes were centrifuged for 10 min at 1,200 × *g* at 4 °C. Plasma was collected in cryovials, put on dry ice and stored at −80 °C until analysis.

### Biochemical analysis

Plasma glucose concentrations were determined on a Roche/Hitachi Cobas c 702-1 analyser using an enzymatic glucose assay (GLUC3, COBAS, Roche Diagnostics GmbH, Mannheim, Germany). A coefficient of variation (CV) of 0.9% was obtained for repeatability (within series), while a CV of 1.3% was obtained for reproducibility (day-to-day). Plasma insulin concentrations were measured using a commercial enzyme-linked immunosorbent assay (ELISA) (catalogue no. 10.1113-10, Mercodia Insulin ELISA, Sweden). The lowest detectable level of insulin was 1.0 mU/L; intra-assay CV: 4% and inter-assay CV: 4%.

### ^13^C isotope recovery in breath

To all test products, 1 mL of stable isotope (10 mg/mL sucrose-^1–13^C (^1–13^C-glucose) (99%-enrichment; Campro Scientific GmbH, The Netherlands (NL)) in tap water) was added. The dosage was based on a pilot test, in which we were able to detect isotope differences in breath. This solution was freshly prepared each testing day, and added with a pipette to the drink or with a syringe to the muffin, approximately 5 min before the subjects consumed the product.

Breath samples were collected by breathing into breath collection bags (F201-VP-5a, FANci2 – HeliFANplus, Leipzig, Germany). Samples were obtained by exhaling through the valve filling the bag to capacity. Two baseline samples were averaged to provide solid baseline ^13^C concentrations, the remaining samples were used to indicate delta over baseline (DOB). Samples were collected before and at 15, 30, 45, 60, 90, 120 and 180 min after the start of consumption of the test food. Samples were stored at room temperature and analysed for ^13^C enrichment in CO_2_ by isotope-selective non-dispersive infrared spectrometry (InfraRed Isotope Spectrometer, IRIS doc, Wagner Analysen Technik GmbH, Bremen, Germany).

### Appetite and ad libitum energy intake

In order to determine subjective appetite, thirst and comfort, we used a questionnaire with six 100-mm visual analogue scales (VAS) to rate hunger, fullness, prospective food consumption, desire to eat, thirst and comfort. The VAS were anchored from ‘not at all’ to ‘very much’.

Four hours after consumption, subjects received an *ad libitum* lunch. The lunch consisted of a mixed meal, containing pasta, minced meat, carrot, onion, bell pepper and a commonly used mixture of macaroni spices from a package (542 kJ/100 g; 38 en% carbohydrates, 36 en% fat, 24 en% protein, 2 en% fibre). The meal was freshly prepared once a week, and then frozen and re-heated per subject in the microwave. At the start, all subjects received 1,800 g on a big plate. Subjects were instructed to eat as much as they wanted until they felt comfortably full. When subjects finished eating, they raised a hand, upon which the meal was removed and they were allowed to read something. They were seated between panels and were asked not to talk to each other or use devices, meaning that distractions were limited. All plates were covertly weighed before and after lunch. Food intake was measured as the difference between the total amount given at the start and after returning the plates. From this, energy and macronutrient intake were calculated.

### Gastrointestinal side effects and evaluation of the test products

Subjects rated the following side effects on a seven-point scale ranging from 1 ‘less than normal’, 4 ‘normal’, to 7 ‘very much more than normal’: bloating, belching, flatulence, nausea and diarrhoea. These questionnaires were completed at baseline, 180 and 270 min after consumption of the test food. Additionally, subjects completed the same questionnaire in their diary before they ate dinner. After the third and fifth test days, subjects were asked to identify the product they consumed via an evaluation questionnaire with multiple choice answers. Of the 15 subjects, 12 completed the questionnaire for the drinks and 13 for the muffins. The D-xylose, L-arabinose and control drink were indicated correctly once, never and six times, respectively. The L-arabinose muffin and the control muffin were indicated correctly six and seven times, respectively.

### Statistical analysis

Data are expressed as means ± standard error of the mean (SEM), except for baseline characteristics, which are expressed as means ± standard deviation (SD). Significance was set at *P* < 0.05. All statistical analyses were performed using SAS software v9.3 (SAS Institute Inc., Cary, NC, USA). Experiments A (fruit-based drinks) and B (muffins) were analysed separately.

Glycaemic and insulinaemic responses were assessed per individual time point and as maximum concentration (C_max_), and time to peak. Incremental area under the curve (iAUC) of plasma glucose and insulin were calculated using the trapezoidal equation, where all points above the baseline value were used for the period ranging from 0 to 180 min. Data were checked for normality by visual inspection of the histograms and Quantile Quantile plot (QQ-plots) of the studentized residuals. All analyses were performed separately for drinks and muffins for the subjects who have completed all treatments (*n* = 15).

A mixed-model analysis (PROC MIXED, SAS) was used to assess the treatment effects on peak (C_max_), time to peak and iAUC for plasma glucose and insulin. ^13^C recovery, *ad libitum* intake and appetite ratings were analysed with the same procedure. Treatment was included as a fixed factor, and subject was included as a random factor. Baseline values of plasma glucose or insulin were added to the model as a covariate. Best-fitted covariance structures were used as per the model; this was autoregressive for the curve parameters and variance components for the individual time points. If main effects were statistically significant, Tukey post hoc tests were conducted on the pre-defined contrasts.

## Results

### Glycaemic and insulinaemic responses

#### Drinks

**Fig. 1:**
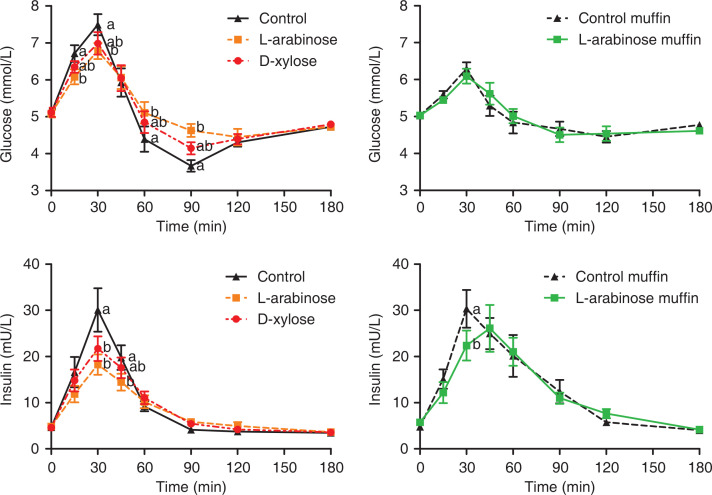
Plasma glycaemic and insulinaemic responses over 180 minutes after consuming the drinks and the muffins in healthy male subjects (*n* = 15). Data are means, with standard error represented by vertical bars. (▲; Control drink), (■; L-arabinose drink), (●; D-xylose drink). (▲; Control muffin), (■; L-arabinose muffin). ^a,b^ different letters indicate significant differences between treatments. To convert insulin in mU/L to pmol/L, multiply by 6.945.

The plasma glucose curve rose sharply and declined quickly for the control drink; however, the rises and declines were less steep for the L-arabinose and D-xylose drinks (Fig. 1). When looking at individual time points, glucose concentrations were lower at 15, 30, 60 and 90 min after the L-arabinose drink, compared with the control drink (treatment [F_(2,322)_ = 0.35, *P* = 0.70]; time [F_(7,322)_ = 108.29, *P* < 0.01]; treatment*time [F_(14,322)_ = 2.83, *P* < 0.01]). Glucose concentrations did not differ between the D-xylose drink and the control drink (all time points *P >* 0.05). The peak concentration of glucose was lower for the L-arabinose and D-xylose drinks compared with the control (C_max_ [F_(2,27)_ = 7.81; *P* = 0.002]) (Table 3). The L-arabinose drink showed a delayed time to peak compared with the D-xylose drink (time to peak [F_(2,29)_ = 4.54, *P* = 0.02]). The iAUC did not differ between drinks (iAUC_0–180 min_ [F_(2,27)_ = 0.51, *P* = 0.61]).

**Table 3:** Plasma glucose and insulin after consumption of a drink or muffin with L-arabinose or D-xylose in healthy men

		A. Fruit-based drinks (*n* 15)						
		Control		L-arabinose		D-xylose		ANOVA: P*
		Mean	SE	Mean	SE	Mean	SE	
Glucose								
C_max_ (mmol/L)		7.6^a^	0.3	6.9^b^	0.2	7.1^b^	0.3	**<0.01**
Time to peak (min)		28^ab^	2	34^a^	2	26^b^	2	**0.02**
iAUC 0–180 min. (mmol × min/L)		75.8	9.2	72.8	14.7	63.3	7.8	0.62
Insulin								
C_max_ (mU/L)		30.5^a^	4.7	19.8^b^	2.3	23.1^ab^	2.8	**<0.01**
Time to peak (min)		31	2	37	3	33	2	0.17
iAUC 0–180 min. (mU × min/L)		890	145	655	117	787	106	0.11

		B. Muffins (*n* 15)						
		Control			L-arabinose			ANOVA: P*
		Mean	SE		Mean	SE		

Glucose								
C_max_ (mmol/L)		6.4	0.2		6.3	0.2		0.49
Time to peak (min)		29	2		31	2		0.45
iAUC 0–180 min. (mmol × min/L)		43.9	8.6		40.9	7.8		0.62
Insulin								
C_max_ (mU/L)		35.1	4.8		30.8	4.9		0.06
Time to peak (min)		39	4		41	3		0.78
iAUC 0–180 min. (mU × min/L)		1,462^a^	213		1,249^b^	182		0.05

* *P*-value from mixed model ANOVA, all drinks were subsequently compared with Tukey adjustment. Values in a row with different superscripts are significantly different.

The plasma insulin curve rose sharply and declined quickly for the control drink, whereas the rises and declines were less steep for the L-arabinose and D-xylose drinks (Fig. 1). When looking at individual time points, insulin concentrations were lower at 30 and 45 min after the L-arabinose drink, compared with the control drink (treatment [F_(2,322)_ = 4.02, *P* = 0.02], time [F_(7,322)_ = 66.05, *P* < 0.001], treatment*time [F_(14,322)_ = 2.48, *P =* 0.002] and Tukey *P* < 0.001 and *P* = 0.04). The peak concentration was also significantly reduced after the L-arabinose drink compared with the control (C_max_ [F_(2,27)_ = 7.01; *P* = 0.004]). After the D-xylose drink, the insulin concentration was significantly lower at 30 min compared with the control drink (Tukey *P* < 0.001; Fig. 1). There were no differences in time to peak or iAUC_0–180 min_ between the drinks [F_(2,27)_ = 2.43; *P* = 0.11] (Table 3).

#### Muffins

The shapes of the plasma glucose curves for the control muffin and the L-arabinose muffin were similar (Fig. 1). However, the plasma insulin curve for the L-arabinose muffin showed a less steep rise than the control muffin (Fig. 1). At 30 min, plasma insulin concentrations were significantly lower (Tukey *P* = 0.01), and the C_max_ and iAUC_0–180 min_ tended to be lower for the L-arabinose muffin compared with the control muffin (*P* = 0.06 and *P* = 0.05, respectively; Table 3). No further differences for glycaemic and insulinaemic responses were observed between the L-arabinose and the control muffin.

### ^13^C recovery in breath

#### Drinks

**Fig. 2:**
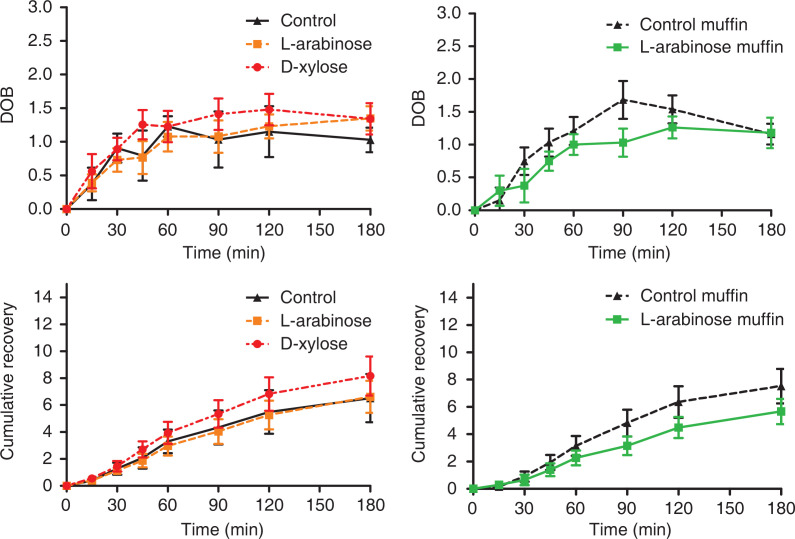
Delta over baseline (DOB) and cumulative ^13^C recovery in breath samples over 180 min. after consumption of the test foods containing either: (▲; Control drink), (■; L-arabinose drink), (●; D-xylose drink). (▲; Control muffin), (■; L-arabinose muffin) in healthy male subjects (*n =* 15). Data are means, with standard error represented by vertical bars.

The DOB was significantly lower for the control and L-arabinose drinks than for the D-xylose drink (treatment *P* = 0.02; time *P* < 0.001; treatment*time *P* = 0.94; Supplementary Fig. S1) (Fig. 2). We also observed this effect in the cumulative recovery; after Tukey adjustment, the L-arabinose drink was overall significantly lower than the D-xylose drink (treatment *P* = 0.02; time *P* < 0.001; treatment*time *P* = 1.00).

#### Muffins

We observed an overall significant treatment effect, where the L-arabinose muffin was significantly lower than the control muffin for DOB (treatment *P* = 0.02; time *P* < 0.001; treatment*time *P* = 0.37), as well as the cumulative recovery (treatment *P* < 0.001; time *P* < 0.001; treatment*time *P* = 0.34; Supplementary Fig. S1) (Fig. 2).

### Appetite and food intake

None of the subjective feelings of appetite (hunger, fullness, desire to eat, prospective food consumption and thirst) were significantly changed when L-arabinose or D-xylose was consumed (Supplementary Table S1 and Supplementary Fig. S2 and S3). Additionally, *ad libitum* intake of the pasta lunch after the test morning was not different between test products (Supplementary Table S2).

### Comfort and gastrointestinal side effects

Feeling of comfort did not differ between the treatments at any time point. The lowest comfort scores were found at baseline, and the highest comfort scores were found after the lunch when the subjects were free to go. Regarding drinks, the mean comfort score at baseline was 58 mm, and after the *ad libitum* lunch, the comfort score was 73 mm after 270 min. Regarding muffins, the mean comfort scores at the start and at the end were similar to the scores for the drinks (62 and 74 mm, respectively). Ratings for bloating, regurgitation, nausea, flatulence and diarrhoea were also not different between test products.

## Discussion

The primary aim of this research study was to compare glycaemic and insulinaemic responses of added L-arabinose to realistic liquid and solid sucrose-containing food products. In order to study this, we used a fruit-based drink and a muffin as test foods. The test foods were similar in available carbohydrate content (i.e. ~50 g). L-arabinose or D-xylose was added at a dosage of 10% w/w of sucrose content to the manipulated foods. Overall, we found clear effects on glucose and insulin responses when L-arabinose and D-xylose were added to the fruit-based drinks. For solid foods, the results were less clear; the addition of L-arabinose did not affect glucose responses after consumption of the muffins; however, at 30 min after consumption, the insulin concentrations were lower compared with the control muffin, and overall, the insulin response tended to be somewhat lower than the control muffin (C_max_ and iAUC_0–180_).

We observed clear significant effects of the addition of 3.5 g L-arabinose to the fruit-based drinks containing 38 g sucrose on glycaemic and insulinemic peaks. The glycaemic peak reduced by 9%, and the insulinaemic peak reduced by 35% compared with the control drinks. These effects are consistent with the results of other studies investigating L-arabinose in sugar-containing drinks (10, 13, 14, 16). One of the studies found that the addition of 2 g L-arabinose to 50 g sucrose in water reduced serum glucose and insulin levels after 30 min (13). Other studies have shown similar results, that is, a significant reduction of the glucose peak with the addition of 1, 2, or 3 g L-arabinose to 75 g sucrose in water (10). Moreover, 2 g (or 5%) L-arabinose with 40 g sucrose in 108 g water lowered blood glucose levels at 15 and 30 min, as well as the blood glucose peak and AUC (14). Additionally, as in this study, the effects on insulin were more pronounced than those on glucose responses; for example, addition of 3 g L-arabinose lowered the insulin peak by 34% and also reduced the iAUC_0–180_ (10). Broadly, glucose and insulin responses decrease as increasing proportions of sucrose are replaced for L-arabinose (16).

The secondary objective of this study was to compare the effects of adding L-arabinose in drinks with those of adding D-xylose in drinks. The results showed that D-xylose showed similar effects on glycaemic responses to L-arabinose. This was consistent with other studies, in which D-xylose was added to sucrose in water (8, 12, 17, 19). For example, Bae et al. tested 5 and 7.5 g D-xylose plus 50 g sucrose in 130 mL water, and found lower glucose and insulin concentrations at 15, 30, and 45 min, as well as an approximately 20% lower AUC of glucose and insulin (12). *In vitro* experiments with L-arabinose have shown that the observed effects can be explained by its uncompetitive inhibition of sucrase activity in the brush border of the intestine, leading to an inhibition of sucrose breakdown and consequently delaying the absorption of glucose (9, 10).

In this study, we also investigated the effect of L-arabinose in a complex solid food matrix, namely, a muffin. The addition of L-arabinose to the muffin did not lower the glycaemic peak or response. However, there was a trend towards a reduced insulin response (significantly lower value at t30 (–26%), and a trend towards a lower C_max_ and iAUC). Similar effects were found, that is, only effects on the insulin response and not on the glucose response, after the intake of ground cereal with 8 or 13% of L-arabinose (16). We speculate that this small effect could have been due to the solid food form but also the more complex nutrient composition. While the drink consisted only of mono- and disaccharides in addition to some flavouring, the muffins also contained fat, starch and protein. It is known that fat and protein affect the rate of gastric emptying (20), and therefore, lower the glycaemic load. More importantly, it may also be that the effect of L-arabinose was overshadowed by the large increase in blood glucose levels due to the starch content in the muffins, as it has been suggested by others that L-arabinose has a similar, but less pronounced, inhibitory effect on maltase activity (15, 21), that is, the enzyme mainly responsible for starch hydrolysis.

In addition to these speculations, the dose of L-arabinose used in this study may have been too low to study its effect on solid food products. However, we added L-arabinose as 10% w/w of sucrose to the products, which was chosen based on a previous study that showed an almost significant effect when a dose of 10% L-arabinose was used in a muffin (15). In liquids, small doses of 4% already enhanced glycaemic responses. However, for solids higher amounts are necessary, as other factors such as nutrient composition and food matrix affect glucose uptake. Therefore, we used the dosage of 10%.

In addition to the effects on glucose homeostasis, we explored the working mechanism of L-arabinose and D-xylose by adding a stable isotope to the test products. These results revealed that adding L-arabinose to sucrose-rich drinks showed no effect on the amount of isotope enriched sucrose that was expired compared with the sucrose drink. The recovery of ^13^CO_2_ was lower after the L-arabinose muffin than after the control muffin. However, there was a difference between L-arabinose and D-xylose in drinks: after consuming the D-xylose drinks, more ^13^CO_2_ was recovered, and it was also more rapidly recovered. The findings in drinks were not in line with our hypothesis, as we expected to find slower or at least a lower level of recovery of ^13^C after the L-arabinose and D-xylose drinks. A study by Sanai et al. has previously demonstrated using a breath test that glucose is metabolised at a much lower rate when ingested together with L-arabinose (22). The results of this study were also not in line with those of the blood glucose and insulin levels. It may be that the relationship between ^13^CO_2_ exhalation and sucrose digestion is not as straightforward as previously thought, and that this method is not suitable for exploration of its working mechanism. Future research should focus further on the metabolism of L-arabinose and D-xylose in the human body.

We did not find any effects of L-arabinose or D-xylose on appetite and spontaneous food intake, although animal studies have shown some effects on body weight (23, 24). We believe that the manipulation in this study was too subtle to reveal differences in feelings of appetite or eating behaviour, as the weights and energy contents of the preloads were similar (25).

Gastrointestinal complaints have been reported after a long-term consumption of L-arabinose (26). We hypothesized that L-arabinose delays sucrose digestion, leading to sucrose and L-arabinose reaching the colon intact, which may then cause gastrointestinal complaints, such as looser stools and bloating. However, in this study, subjects did not report any gastrointestinal symptoms, such as looser stools, bloating and nausea, after consumption of products containing L-arabinose or D-xylose, nor did they drop out due to gastrointestinal symptoms. These observations imply that the dosage of 3.5 g of L-arabinose and D-xylose was acceptable and tolerated by the subjects.

We investigated the effects of L-arabinose and D-xylose in a very homogeneous study population, which included young healthy men with normal weight and normal well-controlled glucose levels. As our primary research question was to test matrix effects, this group was suitable, given their low variation in glucose and insulin responses. As patients with diabetes have problems with regulating their glucose levels, we expect that the glucose and insulin lowering effects will be more pronounced in patients with diabetes. To our knowledge, only one of the studies tested L-arabinose supplementation in metabolic syndrome patients. In these patients, both body weight and waist circumference were decreased after 6 months of daily intake of 20–45 g L-arabinose, as were their fasting glucose levels (26). However, currently the study findings cannot be extrapolated to patients with diabetes. Further studies are needed to investigate these findings in other populations.

In conclusion, the results of this study reveal that the intake of L-arabinose containing drinks results in a clear reduction of plasma glucose response compared with a control drink. Moreover, the glucose and insulin peaks were significantly reduced after the L-arabinose and D-xylose drink compared with the control drink. L-arabinose and D-xylose showed effects of similar size. The intake of muffins did not lead to a similar effect on insulin responses; however, we observed a significantly reduced level of insulin after 30 min and a trend towards a lower iAUC. None of the treatments led to effects on feelings of appetite, wellbeing or gastrointestinal comfort or *ad libitum* energy intake.

In this study, we provide important knowledge on the implementation of L-arabinose addition in real-life foods rich in sucrose to improve blood glucose control as this information is currently lacking. It appeared that the matrix is crucial for its functionality; it was more difficult to study the effect of L-arabinose on solid food products, and dosages higher than 10% of L-arabinose of sucrose may be needed. Further studies are warranted to better understand the addition or replacement of L-arabinose in other sucrose-rich food products with different nutrient compositions and textures. Moreover, the metabolic route of L-arabinose in the human body should be investigated. Finally, the effect of L-arabinose should be studied in a more heterogeneous population, especially in people at risk of developing type 2 diabetes, such as pre-diabetics and glucose-intolerant patients.

## Data Availability

Available upon request.

## Trial registration

The trial was pre-registered in the Dutch Trial Register (NL4818/NTR5319, Netherlands Trial Register, www.trialregister.nl) on 15 April 2015.

## Code availability

SAS syntaxes of analyses are available upon request.
